# Effect of doxofylline on pulmonary inflammatory response and oxidative stress during mechanical ventilation in rats with COPD

**DOI:** 10.1186/s12890-022-01859-6

**Published:** 2022-02-17

**Authors:** Zhi-yuan Chen, Yu-mei Lin, Jian-hua Wu, Xiao-qi Zhang, Yi Zhang, Wen-xi Xie, Shu-qiang Chu, Yan Li

**Affiliations:** 1 Department of Anesthesiology, The Second Affiliated Hospital of Fujian Medical University, No. 950 of Donghai Street, Fengze District, Quanzhou, 362000 China; 2 Department of Pathology, The Second Affiliated Hospital of Fujian Medical University, Quanzhou, 362000 China

**Keywords:** COPD, Doxofylline, Inflammatory response, Oxidative stress, PaO_2_, PaCO_2_

## Abstract

**Objective:**

To evaluate the effects of doxofylline on inflammatory responses and oxidative stress during mechanical ventilation in rats with chronic obstructive pulmonary disease (COPD).

**Methods:**

Eight-week-old male Sprague Dawley rats were selected, and the COPD rat model was constructed. The rats were randomly divided into a model group (group M), a model + normal saline group (group N), a doxofylline group (group D), and a control group fed with conventional chow and given normal oxygen supply (group C) (n = 12 in each group). Tracheal intubation and mechanical ventilation were conducted in the rats in each group after anesthesia. A real-time intravenous infusion with 50 mg/kg of doxofylline was conducted in group D, and there was no drug intervention in groups C, N and M. Pathological manifestations of the pulmonary tissues were observed and compared among the groups. And some indicators were evaluated.

**Results:**

(1) The pulmonary tissues of the rats in groups M, N, and D exhibited typical pathological histological changes of COPD. (2) Groups M, N, and D showed increased Ppeak, PaCO_2_, total white blood cell count in BALF, and IL-8, TNF-α, and MDA levels in the pulmonary tissue and BALF, and decreased PaO_2_ and IL-10 and SOD levels, compared with group C. (3). Group D showed decreased Ppeak, PaCO_2_, total white blood cell count in BALF, and IL-8, TNF-α, and MDA levels in the pulmonary tissue, and increased PaO_2_ and IL-10 and SOD levels, compared with group N or M.

**Conclusion:**

Doxofylline was shown to improve ventilation and air exchange during mechanical ventilation in rats with COPD, reduce the inflammatory response and oxidative stress, and mitigate the degree of pulmonary tissue injury.

## Introduction

Doxofylline is a methylxanthine derivative of a phosphodiesterase inhibitor, and, as a new type of theophylline, it has been proven to exhibit superior pharmacological effects to standard theophylline [1]. Intravenous administration of doxofylline can rapidly counteract the constricting effect of adenosine on the respiratory tract, dilate the bronchi, and inhibit the severity of inflammatory factors, thus providing an anti-inflammatory effect [2]. Furthermore, doxofylline can inhibit the release of endogenous catecholamines and calcium, promote the movement of airway cilia, reduce spasm of the airway smooth muscle, strengthen diaphragm contraction, and help improve the pulmonary ventilation function [3].

Chronic obstructive pulmonary disease (COPD) is a preventable and treatable disease, characterized by airflow limitation that is not fully reversible. The condition may be progressive and is correlated with abnormal chronic inflammation of the airways and pulmonary tissue in response to harmful gases or particles such as cigarette smoke. The pathological changes are mainly manifested as chronic bronchitis and symptoms similar to emphysema. The main pathophysiological change is persistent airflow restriction, leading to pulmonary ventilation dysfunction. With the development of COPD, dysfunction of ventilation and air exchange may cause hypoxia and carbon dioxide retention, and eventually lead to respiratory failure [4]. Chronic inflammation, protease/anti-protease imbalance, and oxidative and antioxidant imbalance are all involved in the pathogenesis of COPD [5, 6]. Mechanical ventilation during surgical anesthesia tends to result in elevated airway pressures, causing an imbalance in the ventilation/blood flow ratio in the lungs and a release of inflammatory mediators.

Based on the above pharmacological effects of doxofylline and the pathological characteristics of COPD, it can be assume that rats with COPD aggravate the inflammatory response and oxidative stress imbalance during mechanical ventilation. The intervention of doxofylline can not only improve ventilation, but also and further reduce the pulmonary inflammatory response and oxidative stress imbalance in rats with COPD, thereby improving lung function. In the present study, the protective effects of doxofylline on pulmonary function in mechanically ventilated COPD rats were investigated in terms of the inflammatory response and oxidative stress.

## Materials and methods

### Apparatus

The apparatus used was as follows: Reward R407 small animal ventilator (Shenzhen Reward Life Science & Technology Co., Ltd., China); Image Quant LAS4000 mini imager (GE, USA); Image Pro Plus 6.0 image analysis system (Media cybernetics Co., USA); and Radox blood gas analyzer ABL80 (RADIOMETER, Denmark).

### Drugs and reagents

The drugs and reagents utilized were as follows: doxofylline infusion solution (batch number: 20160115, Jiangsu Enhua Pharmaceutical Co., Ltd.); Fujian tobacco (China Tobacco Industry Co., Ltd., of Fujian Province); bacterial lipopolysaccharide (LPS) (batch number: L2880-10MG, Sigma Co., USA); rat tumor necrosis factor α (TNF-α), interleukin (IL)-8, IL-10 enzyme-linked immunosorbent assay (ELISA) test kit (Shanghai Youyou Biotechnology Co., Ltd.); malondialdehyde (MDA) detection kit A003-1 (thiobarbituric acid [TBA] method, Nanjing Jiancheng Institute of Biological Engineering); superoxide dismutase (SOD) detection kit (Nanjing Jiancheng Institute of Biological Engineering); and blood gas analysis test card (SC80 BASIC).

### Rat selection and number estimation

A total of 64 clean-grade (CL), male, eight-week-old Sprague Dawley (SD) rats, each weighing 200–280 g, were purchased from Fuzhou Minhou County Wu's Laboratory Animal Trade Co. (laboratory animal certificate number: SCXK(Hu)2012-0002). The experimental animals were kept in the Laboratory Animal Center of Quanzhou Medical College, and they were all kept and handled following the guidelines for the management and application of laboratory animals. In the pre-experimental stage, the survival rate of rats in the COPD model construction group was approximately 85% during the feeding process. To randomly select the required number of rats from the surviving specimens, the numbers of rats in the conventional feeding group with normal oxygen and in the model construction group were increased in equal proportion during the model construction, and the remaining rats that were not randomly selected for the experiment were kept normally until natural death.

### Construction of COPD rat model

The sample of 64 eight-week-old male SD rats was first randomly divided to place 16 rats in the conventional chow and normal oxygen supply group and 48 in the model construction group. The rats in the conventional chow and normal oxygen supply group were reared under normal oxygen for 60 days, with endotracheal injection of normal saline conducted on the 1st and 30th days. The rats in the model construction group were exposed to smoke from three lit, unfiltered cigarettes for 30 min per day for 60 days in a homemade 4 L glass resin container, with reference to the methods described by Tang et al. [7] and Chen et al. [8]. Endotracheal injection of 200 μg of 0.2-ml-volume bacterial LPS was conducted on the 1st and 30th days.

### Rat grouping, mechanical ventilation, and drug intervention

After 60 days, 36 rats were randomly selected from the surviving rats in the model construction group and randomly divided into three groups of 12: a model group (group M), a model + normal saline group (group N), and a doxofylline group (group D). Twelve rats from the conventional chow and normal oxygen supply group were randomly selected as the control group (group C). The rats in each group were first intraperitoneally anesthetized with 1% pentobarbital sodium (40 mg/kg), and then the right femoral vein was intubated together with tracheal intubation. Following the method described by Li et al. [9], the small animal ventilator was connected to conduct mechanical ventilation for 120 min, and the relevant parameters were set as follows: tidal volume = 8 ml/kg; inspiration-to-expiration ratio = 1:1; respiratory rate = 80 times/min; inhaled oxygen concentration = 0.5; and positive end-expiratory pressure = 0. After tracheal intubation, a real-time intravenous infusion of 50 mg/kg of doxofylline (dissolved in 0.3 ml of normal saline) was applied to the rats in group D, following Rao et al.'s [10] method, and an equal volume of normal saline was given to the rats in groups C and N. The animal experimentation was approved by the Experimental Animal Ethics Committee of Fujian Medical University, with the approval number NO.37, 2018.

### Blood gas analysis and detection of arterial partial pressure of carbon dioxide and arterial partial pressure of oxygen

In each group of rats, after 120 min of mechanical ventilation, the abdominal wall was incised under anesthesia, and the abdominal aorta was isolated and exposed**.** A G22 arterial puncture needle was used to puncture the abdominal aorta, and 1 ml of arterial blood was obtained. The animals were executed by blood release. Arterial partial pressure of carbon dioxide (PaCO_2_) and arterial partial pressure of oxygen (PaO_2_) were quickly measured using a blood gas analyzer.

### Histopathological examination of pulmonary tissue (hematoxylin and eosin staining)

In this experiment, lung tissue pathological section was stained with HE to evaluate the pathological changes of rat lung tissue. For each rat, the thoracic and abdominal cavities were fully exposed, and then the right middle lobe of the lung was separated and placed in 10% formalin solution for fixation. The tissue was paraffin-embedded and then serially sectioned into three sets. One set of the pulmonary tissue sections was taken for routine hematoxylin and eosin staining and analysis to observe histopathological changes of the lung.

### Detection of peak airway pressure at various time points during mechanical ventilation

Peak airway pressure (Ppeak, cmH_2_O) was monitored by a small animal ventilator at the following time points: immediately after tracheal intubation (T0); and at 30, 60, and 120 min after drug injection (T1, T2, and T3, respectively).

### Determination of interleukin-8, -10, and tumor necrosis factor α (TNF-α) in the pulmonary tissue by enzyme-linked immunosorbent assay

The rat IL-8, IL-10, and TNF-α ELISA kits and the required reagents were used for the detection. The right lower pulmonary tissue in the rat was removed from the lyophilization tube with the preparation of the tissue homogenate. The standard solution was prepared, and then the sample was added and the plate was washed, with the successive addition of the first antibody working solution, the enzyme-labeled antibody working solution, the substrate working solution, and the termination solution. The absorbance was measured at 450 nm with an enzyme marker.

### Determination of total white blood cell count and concentrations of IL-8, IL-10, and TNF-α in the bronchoalveolar lavage fluid

With thoracotomy, the left main bronchus was intubated with an epidural puncture needle, and 5 ml of normal saline at 4℃ was slowly injected. The swollen lung was gently massaged for approximately 1 min, the solution was pumped back and re-injected, and the lavage was repeated three times. From this process, the total recovery rate of bronchoalveolar lavage fluid (BALF) was > 80%, and the product was taken as the BALF specimen. The recovered BALF was mixed, and 0.5 mL was first taken to count the cells with a cell-counting plate. The remaining liquid was centrifuged at 4℃, at a rate of 1,000 r/min, for 10 min, and the supernatant was taken and stored at − 20℃. The concentrations of IL-8, IL-10, and TNF-α in the BALF were measured by ELISA with BALF supernatant. The operation procedure was conducted in strict accordance with the kit instructions.

### Determination of the concentrations of MDA and SOD in the pulmonary tissue

The MDA content in the pulmonary tissue homogenate was determined by applying the TBA method in the sectioned right lower pulmonary lobe. The basic principles of this method are as follows: TBA can have a condensation reaction with MDA in peroxidized lipid degradation products, resulting in a red product, which has a maximum absorption peak of 532 nm, and OD can be measured by spectrophotometer; then, the MDA content in the measured sample can be calculated using a formula. The SOD content was determined using the xanthine oxidase method (transamine method). This method is based on the following processes: xanthine oxidase catalyzes xanthine to produce superoxide anion radicals, which oxidize hydrochloric acid by amine into nitrite; as a result, nitrate and p-aminobenzenesulfonic acid and methylnaphthylamine may demonstrate a purple–red color, which has a maximum absorption peak at 550 nm, and the absorbance can be measured by a visible light spectrophotometer. If the measured sample contains SOD, it has a specific inhibition effect on the superoxide anion radical; thus, the nitrite that can be formed is reduced, and the absorbance of the measured tube is lower than that of a blank tube during the colorimetric measurement, and the vitality of SOD in the measured sample can be calculated by the formula.

### Statistical analysis

The software package SPSS 20.0 was used for data analysis. After the normality test and chi-square test, the results of the measurable data were expressed as means ± standard deviations (${\overline{\text{x}}}$ ± s). One-way analysis of variance (ANOVA) was used for the comparisons between groups, while ANOVA with repeated measures was used to compare the values within groups at different time points. A *P* value of < 0.05 was considered statistically significant.

## Results

### General characteristics and histopathological manifestations of rats in each group

**Fig. 1:**
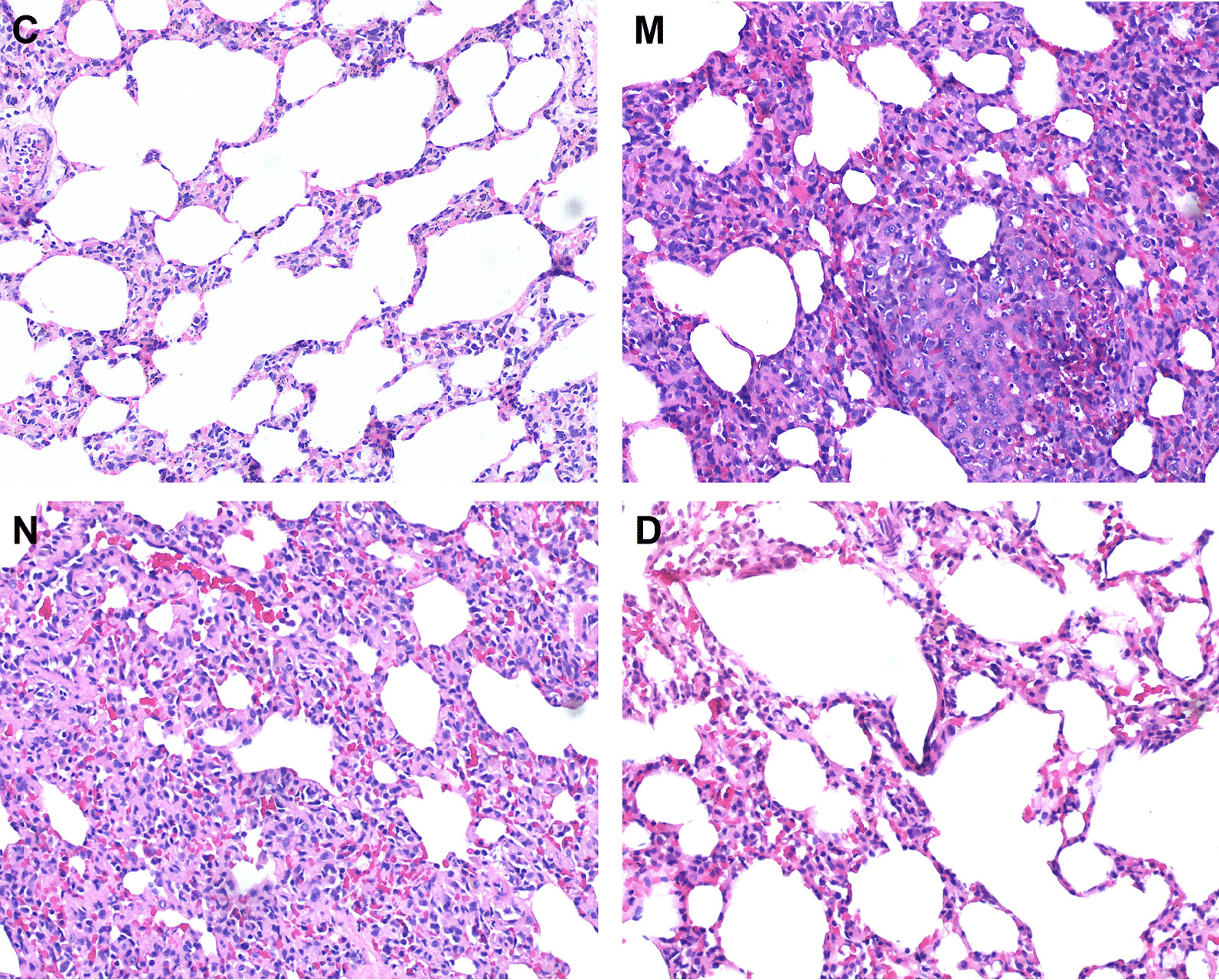
Images of the pulmonary histopathological sections in various groups of rats (HE staining × 100). Model (group M) + normal saline group (group N) doxofylline group (group D). Control group (group C)

The rats in group C were in better general condition than those in the COPD model: they were more lively and active, and showed better hair luster, smooth and regular respiration, and rapid weight gain. The rats in the COPD model group showed reduced activity, gradual loss of hair luster and tiredness, and shortness of breath. Other symptoms developed in the later stages of the model construction, with slight activity and slow weight gain. The results of light microscopy showed that the pulmonary tissue of the rats in group C had normal small airway mucosa structures, with no obvious inflammatory cell infiltration, and normal alveolar cavity size. The pathological histology of the rats in group M showed significantly proliferated and hypertrophied mucosal cup cells and glands, alongside the bronchial mucosal folds increasing, becoming longer, and protruding into the lumen. Inflammatory cells, mainly consisting of macrophages (dust cells) and lymphocytes, were observed in the mucosal and submucosal layers of the bronchi at all levels, in addition to plasma cells and a small number of neutrophils, and mucus plugs and a large number of neutrophils were visible in the bronchial lumen. A large number of macrophages engulfed with smoke particles were visible in the bronchus and the pulmonary arterioles, and some macrophages were disintegrated and destroyed. The distal end of the terminal bronchiole narrowed, the respiratory bronchioles and alveolar ducts were cystically expended, the alveolar cavity was irregularly enlarged, and the alveolar walls were thinned, swollen, ruptured, or fused to form bullae, all of which are in line with the typical histopathological changes of COPD. There were no significant differences between the histological changes in groups M and N. Group D showed reduced small airway mucosal edema and inflammatory cell infiltration compared with group N. Mild sub-mucosal gland hyperplasia and hypertrophy were visible in group D, and the number of alveoli was reduced, with a few alveoli ruptured and fused to form bullae. These findings are presented in Fig. 1.

### Ppeak at different time points in rats in each group

**Fig. 2:**
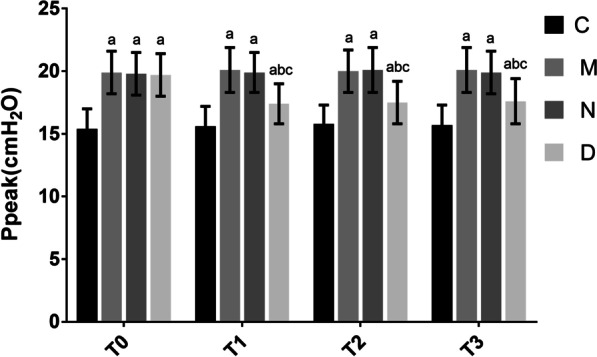
Comparison of Ppeak (cmH_2_O) at different time points among the different groups of rats (n = 12, $\overline{X}$ ± s). Note: Compared with group C, ^a^*P* < 0.05; compared with group N, ^b^*P* < 0.05; compared with T0 in group D, ^c^*P* < 0.05

There were no significant differences between Ppeak values at T0 for groups M, N, and D (*P* > 0.05). Groups M, N, and D showed significantly higher Ppeak than group C at each time point (*P* < 0.05). There were no significant differences between Ppeak for groups M and N at any time point (*P* > 0.05). Group D showed significantly decreased Ppeak compared with group N at T1, T2, and T3 (*P* < 0.05). Within group D, Ppeak was significantly reduced at T1, T2, and T3 compared with T0 (*P* < 0.05). These findings are presented in Fig. 2.

### Comparison of PaCO_2_ and PaO_2_ among different groups

**Fig. 3:**
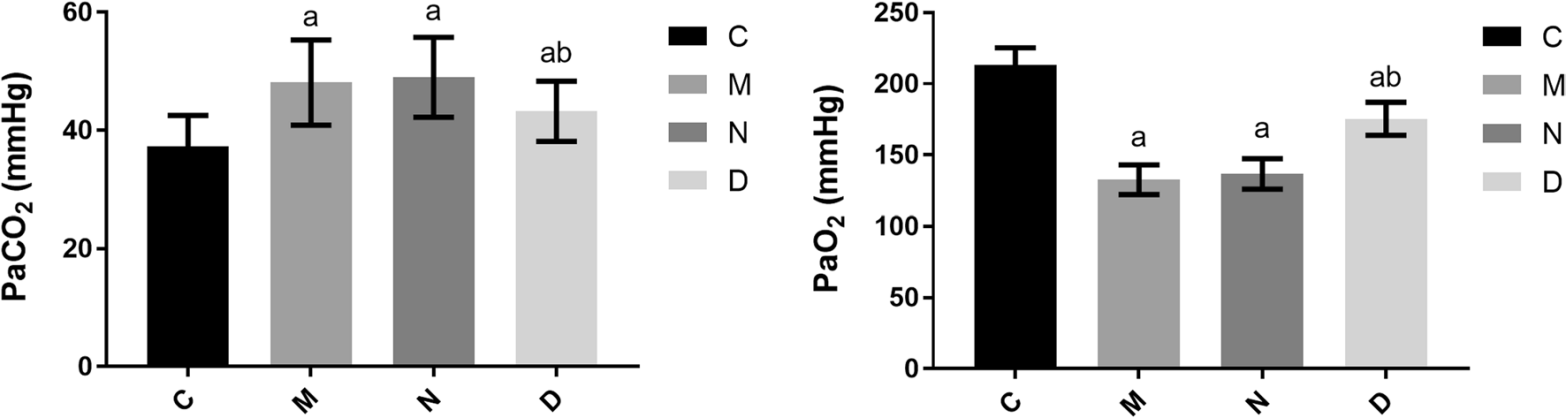
Comparison of PaCO_2_ and PaO_2_ in blood gas analysis among the different groups of rats (n = 12, $\overline{X}$ ± s). Note: Compared with group C, ^a^*P* < 0.05; compared with group N, ^b^*P* < 0.05

Groups M, N, and D showed significantly higher PaCO_2_ (*P* < 0.05) and significantly lower PaO_2_ (*P* < 0.05) levels than group C. There were no significant differences in PaCO_2_ and PaO_2_ between groups M and N (*P* > 0.05). Group D showed significantly decreased PaCO_2_ (*P* < 0.05) and significantly increased PaO_2_ (*P* < 0.05) compared with group N. These findings are presented in Fig. 3.

### Comparison of total white blood cell count and concentrations of inflammatory mediators in BALF among rats in different groups

**Fig. 4:**
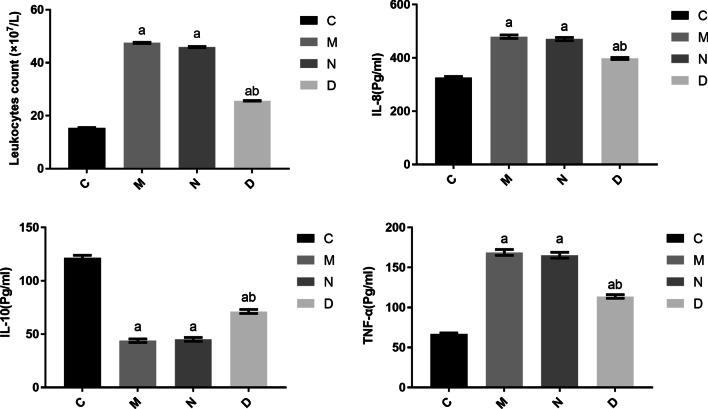
Comparison of total white blood cell count and IL-8, IL-10, and TNF-α levels in BALF among the different groups of rats (n = 12, $\overline{X}$ ± s). Note: Compared with group C, ^a^*P* < 0.05; compared with group N, ^b^*P* < 0.05

Groups M, N, and D showed significantly increased total white blood cell count and levels of IL-8 and TNF-a in BALF (*P* < 0.05), and significantly decreased levels of IL-10 (*P* < 0.05), compared with group C. There were no significant differences in total white blood cell count or levels of IL-8, IL-10, or TNF-α between groups M and N (*P* > 0.05). Group D showed significantly decreased total white blood cell count and levels of IL-8 and TNF-α in BALF (*P* < 0.05), and significantly decreased IL-10 levels (*P* < 0.05), compared with group N. These findings are presented in Fig. 4.

### Comparison of inflammatory mediators in the pulmonary homogenate among rats in different groups

**Fig. 5:**
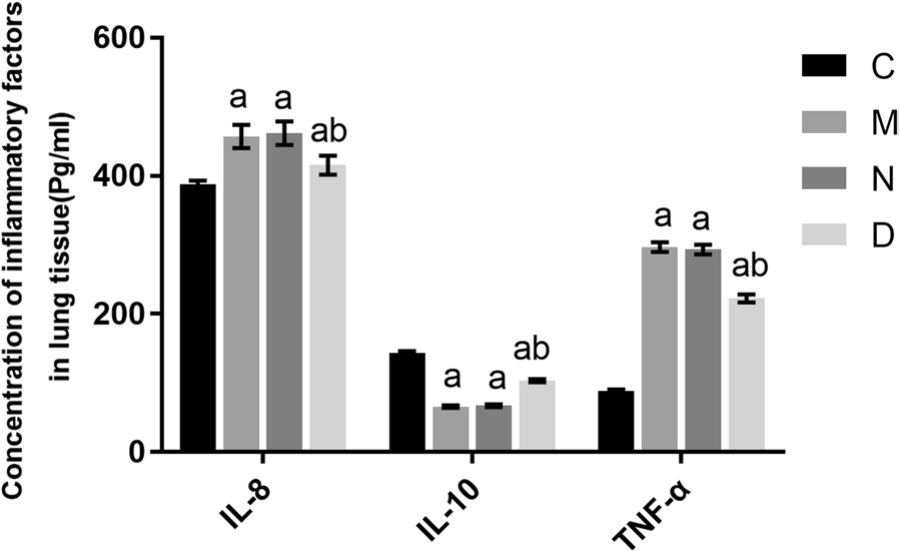
Comparison of IL-8, IL-10, and TNF-α levels in the pulmonary tissue among the different groups of rats (n = 12, $\overline{X}$ ± s). Note: Compared with group C, ^a^*P* < 0.05; compared with group N, ^b^*P* < 0.05

Groups M, N, and D showed significantly increased IL-8 and TNF-α levels (*P* < 0.05), and significantly decreased IL-10 levels (*P* < 0.05), compared with group C. There were no significant differences in IL-8, IL-10, or TNF-α levels between groups M and N (*P* > 0.05). Group D showed significantly decreased IL-8 and TNF-α levels (*P* < 0.05), and significantly increased IL-10 levels (*P* < 0.05), compared with group N. These findings are presented in Fig. 5.

### Comparison of MDA and SOD contents in the pulmonary homogenate among rats in different groups

**Fig. 6:**
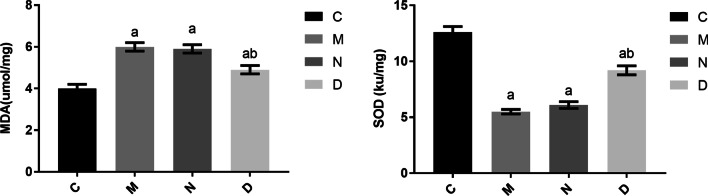
Comparison of MDA and SOD levels in the pulmonary tissue among the different groups of rats (n = 12, $\overline{X}$ ± s). Note: Compared with group C, ^a^*P* < 0.05; compared with group N, ^b^*P* < 0.05

Groups M, N, and D showed significantly increased MDA content (*P* < 0.05) and significantly decreased SOD content (*P* < 0.05) compared with group C. There were no significant differences in MDA or SOD content between groups M and N (*P* > 0.05). Group D showed significantly decreased MDA content (*P* < 0.05) and significantly increased SOD content (*P* < 0.05) compared with group N. These findings are presented in Fig. 6.

## Discussion

The pathological basis of COPD is a chronic inflammation of the airway walls and pulmonary parenchyma together with emphysema [11]. The ideal animal COPD model should have common etiologies consistent with human disease and histopathological changes characterized by airway remodeling and emphysematous pathology, which should be consistent with clinical practice. Cigarette smoke exposure offers the closest environment to the disease in humans, and this method has been successfully adopted in the construction of various animal COPD models [12]. In the evaluation of animal COPD models, pulmonary histopathological examination can determine whether the pathological changes in the small airways and pulmonary parenchyma are consistent with the typical changes seen in COPD [13]. In the present experiment, the COPD rat model was constructed by fumigation and intratracheal injection of LPS. The histopathological results of the rat lungs showed severe deformation of the bronchial lumen, marked detachment of the bronchial mucosa epithelium, cilia inversion, cupped cell hyperplasia, hyperplasia of submucosal glands, and a large number of inflammatory cells infiltrating around the duct wall, together with alveolar wall thinning and alveolar lumen enlargement and fusion into the pulmonary bullae, all representing typical pathological changes of COPD. According to our findings, Ppeak values during mechanical ventilation and PaCO_2_ levels in the arterial blood gas analysis were significantly higher in groups M, N, and D than in group C. These results suggest the existence of pathological changes relating to continuous airflow obstruction in the small airways in the COPD model group, indicating that the model was successfully constructed.

Narrowing of the small airways in COPD patients caused by chronic airway inflammation and airway remodeling often results in increased ventilation resistance during mechanical ventilation, and excessive ventilation pressure often leads to pressure injury to the pulmonary tissue and a release of bio-inflammatory substances [14]. In the present study, following the existing literature, an intervention of femoral vein injection of doxofylline was conducted in the rats in group D [10]. The ventilation pressure of the rats in this group decreased significantly during mechanical ventilation from 30 min after the intravenous injection, continuing to 120 min after injection. The arterial blood sample was collected after 120 min of mechanical ventilation, and PaCO_2_ and PaO_2_ were measured using a blood gas analyzer. The results showed significantly lower PaCO_2_ in the rats in group D than in group N, while PaO_2_ was significantly higher in group D than in group N. These findings could be due to doxofylline reducing the ventilation pressure and improving ventilation by inhibiting phosphodiesterase activity in the smooth muscle cells and regulating intracellular cyclic adenosine phosphate content, thus promoting smooth muscle relaxation and bronchodilation [15].

IL-8 is a multicellular-derived cytokine that has a chemotaxis effect allowing the inflammatory cells, especially the neutrophils, to reach the site of inflammation, thus inducing activation of neutrophils, causing an increase in the number and shape of peripheral blood neutrophils, promoting degranulation, producing respiratory bursts, releasing superoxide dismutase and lysosomal enzymes, and aggravating the inflammatory response [16]. TNF-α is a monoclonal factor, produced mainly by monocytes and alveolar macrophages, that has a variety of pro-inflammatory mediator functions, including promoting the adhesion of inflammatory cells, chemotaxis, and infiltration. These processes can rapidly result in lung injury and the release of large amounts of reactive oxygen radicals, protein hydrolases, lipid mediators, and cytokines [17]. IL-10 is the most important anti-inflammatory cytokine in the body, and it plays a particularly prominent role in reducing immune-mediated inflammation. IL-10 inactivates macrophages by inhibiting the production of interferon γ (INF-γ), IL-2, etc., and further development of inflammation leads to immunosuppression and reduction of the inflammatory response [18]. In chronic airway lesions in COPD, inflammatory cells such as the neutrophils, alveolar macrophages, and T lymphocytes show increased exudation and release various inflammatory mediators, including IL-8, TNF-α, and IL-10 [19]. These inflammatory mediators form a complex network system, which leads to the accumulation and infiltration of large numbers of inflammatory cells in the pulmonary tissue, repeated and alternating injury and repair in the lung, progressive and irreversible restriction of the airflow, injury to the alveolar wall, and fibrosis of the pulmonary tissue [20]. In the present study, the total white blood cell count in BALF and the concentrations of IL-8 and TNF-α in BALF and pulmonary tissues were significantly higher, while the concentration of IL-10 was significantly lower, in groups M, N, and D than in group C, suggesting the existence of an inflammatory response in the pulmonary tissues in the COPD rat model. After the intervention treatment with doxofylline, total white blood cell count in BALF and the concentrations of IL-8 and TNF-α in BALF and pulmonary tissues in group D were significantly lower, while the concentration of IL-10 was significantly higher, than in group N, indicating that doxofylline could reduce the pulmonary inflammatory response during mechanical ventilation in rats with COPD.

As a risk factor for COPD, cigarette smoke significantly increases the burden from oxidants and greatly increases the potential for oxidative stress injury [21]. The MDA produced by oxidative stress has been widely studied as a peroxidation product, and increased levels have been detected in patients with asthma and COPD [22]. The degree of peroxidation response in rats can be assessed by observing the MDA level in a COPD rat model [16]. The results of the present study showed that the levels of MDA in the pulmonary tissue in rats in groups M, N, and D were significantly higher than those in group C, indicating an increased level of oxidative stress in COPD rats. After the intervention with doxofylline, the expression level of MDA in the pulmonary tissue in the rats in group D was significantly lower than that in group N. This indicates that doxofylline could reduce the level of MDA in COPD rats and alleviate the pulmonary tissue cell injury caused by excessive oxidative stress, thus protecting the pulmonary tissue.

Oxygen radicals are disproportionated by SOD to produce O_2_ and H_2_O_2_. The latter can be further converted to H_2_O and O_2_ by catalase (CAT), or to hydroxyl compounds and water by glutathione peroxidase-1 (GPx-1). The harmful oxygen radicals are converted to harmless O_2_ and H_2_, etc., with the help of SOD, CAT, etc., thus maintaining the homeostasis of the intracellular environment [23]. SOD has an antioxidant effect and is the primary substance for scavenging oxygen free radicals in the body; thus, the antioxidant ability of the body can be evaluated by observing changes of SOD levels [24]. The results of the present study revealed decreased SOD activity in the pulmonary tissues in groups M, N, and D, indicating that the pulmonary tissues in the COPD rats had a reduced ability to locally destroy the free radicals generated by various oxidative reactions and a reduced antioxidant capacity, resulting in an oxidative/antioxidant imbalance. The level of SOD activity in the pulmonary tissue in group D was higher than that in group N, indicating that doxofylline intervention could enhance SOD activity in mechanically ventilated COPD rats and thus protect the pulmonary tissue from the perspective of maintaining oxidative stress balance. There are still some limitations in this study, which did not consider the effect of the drug on the cell model. In future experiments, by establishing COPD in vitro cell models, we will provide a reliable technology platform for COPD mechanism research, drug screening and research and development.

## Conclusion

In the present study, a COPD rat model was adopted to investigate the effects of doxofylline on pulmonary function, inflammatory response, and oxidative stress in mechanically ventilated COPD rats. After doxofylline intervention, Ppeak was reduced in the mechanically ventilated COPD rats, while ventilation and air exchange efficiency were improved. Reductions in the levels of inflammatory response and oxidative stress were also observed, resulting in mitigation of pulmonary tissue injury.

## Data Availability

We declared that materials described in the manuscript, including all relevant raw data, will be freely available to any scientist wishing to use them for non-commercial purposes, without breaching participant confidentiality.
