# Editorial Expression of Concern: Effect of doxofylline on pulmonary inflammatory response and oxidative stress during mechanical ventilation in rats with COPD

**DOI:** 10.1186/s12890-024-03049-y

**Published:** 2024-05-14

**Authors:** Zhi-yuan Chen, Yu-mei Lin, Jian-hua Wu, Xiao-qi Zhang, Yi Zhang, Wen-xi Xie, Shu-qiang Chu, Yan Li

**Affiliations:** 1 Department of Anesthesiology, The Second Affiliated Hospital of Fujian Medical University, No. 950 of Donghai Street, Fengze District, Quanzhou, 362000 China; 2 Department of Pathology, The Second Affiliated Hospital of Fujian Medical University, Quanzhou, 362000 China

**Correction: BMC Pulmonary Medicine (2022) 22:66**

10.1186/s12890-022-01859-6

The Editor would like to notify readers that concerns have been raised regarding the data in this article. Specifically, panel N of Fig. 1 appears to be duplicated from a previously published article from the same authorship group [1]. Readers are advised to interpret the results with caution.

Zhi-yuan Chen has stated on behalf of all authors that they agree with this Editorial Expression of Concern.

## References

[1] Liu CY, Wu JH, Chen ZY, Zhang Y, Huang CL, Lin AM, Xu XT, Gao XH. Effect of Doxofylline on reducing the inflammatory response in mechanically ventilated rats with chronic obstructive Pulmonary Disease. Int J Chron Obstruct Pulmon Dis. 2021;16:2375–83. 10.2147/COPD.S315639. 34429595 10.2147/COPD.S315639 PMC8378927

